# Two Clinical Cases

**Published:** 1889-09

**Authors:** Clarence N. Payne

**Affiliations:** Port Jervis, N. Y.


### TWO CLINICAL CASES.

Clarence N. Payne, M. D., Port Jervis, N. Y.

(1.) On October 6th, 1888, I prescribed Apis$^3$ for the following symptoms in a little girl of six years of age, light hair and blue eyes:

No appetite; nausea; is very weak; vertigo; headache in temples; no thirst, but water tastes like iron-rust; dislike for sweet things; abdomen very tender < by walking. Feels best in middle of day; drowsy in P. M. Had passed no urine for twenty-four hours. Temperature 100°.

Saw patient two days after this, when there was great improvement in every way, but the following symptoms had developed, viz.: intense itching < on hands and feet and at night; no eruption visible. ℞ Sul.$^{30}$.

Four days later, October 12th, last symptom was no better; itching very severe. At times skin has red blotches (perhaps due to scratching) < at night and only relieved by cold water and then for only a short time. Would like to keep hands and feet in water all the time.

This seemed very much like Apis, and I gave it in the 200th potency. Result, much relief in a few hours, and needed no more medicine after Apis$^{200}$.

*Query.* Were the skin symptoms as above produced by Apis$^3$, and if so, did Apis$^{200}$ cure symptoms produced by same remedy in lower potency? It would seem so.

(2.) Baby K., age ten months, on July 24th, 1889, had suffered with diarrhœa for several days, with three to ten movements per day, of following character: thin, watery, profuse, painless; in color almost like water; very offensive; some excoriation; no flatulence. Prolapsus of rectum during movement. ℞ Podo.$^3$.

July 25th.—Prolapsus better, but no improvement or change in condition of movements. Baby feels well, is playful. Is no weaker or lighter in weight in spite of large number of movements. ℞ Phos-ac.$^{200}$.

July 26th.—No improvement or change. ℞ Phos-ac.$^3$.

July 27th.—Better, less number movements, continued to improve and cured under use of Phos-ac.$^{3}$.

In this case the 3d potency seemed to act better than the 200th.

